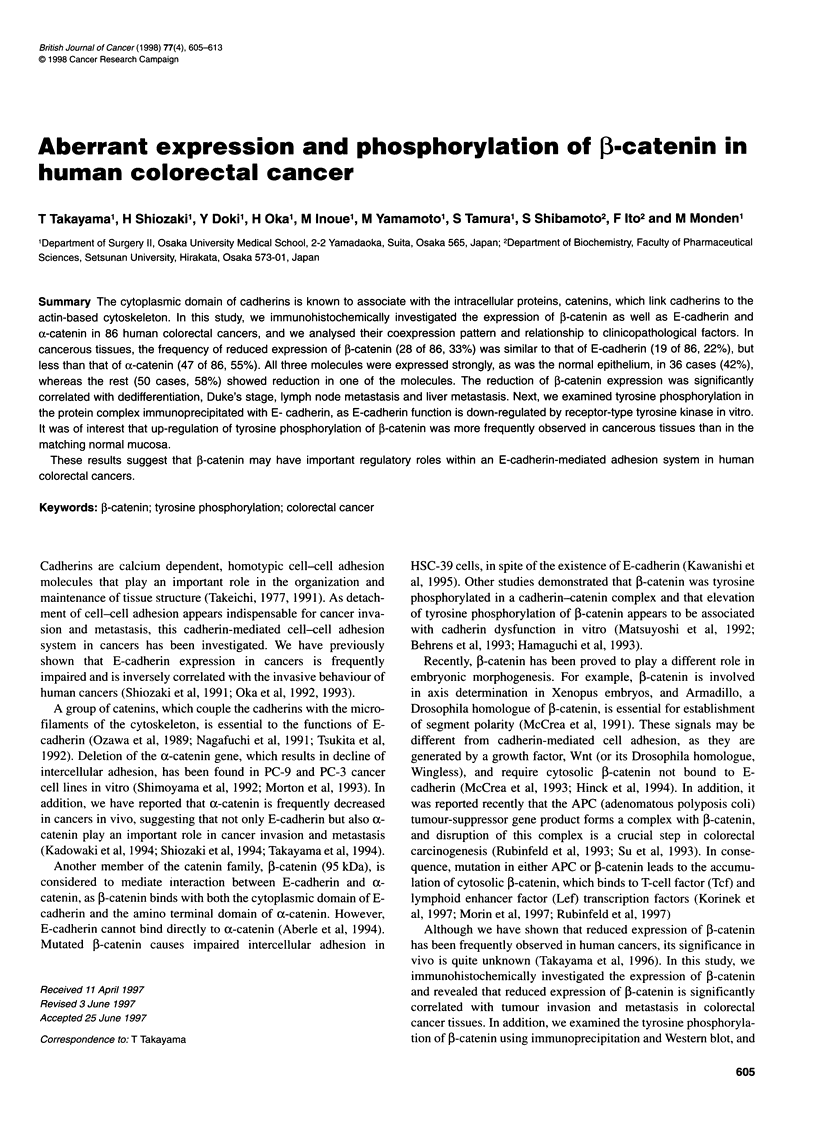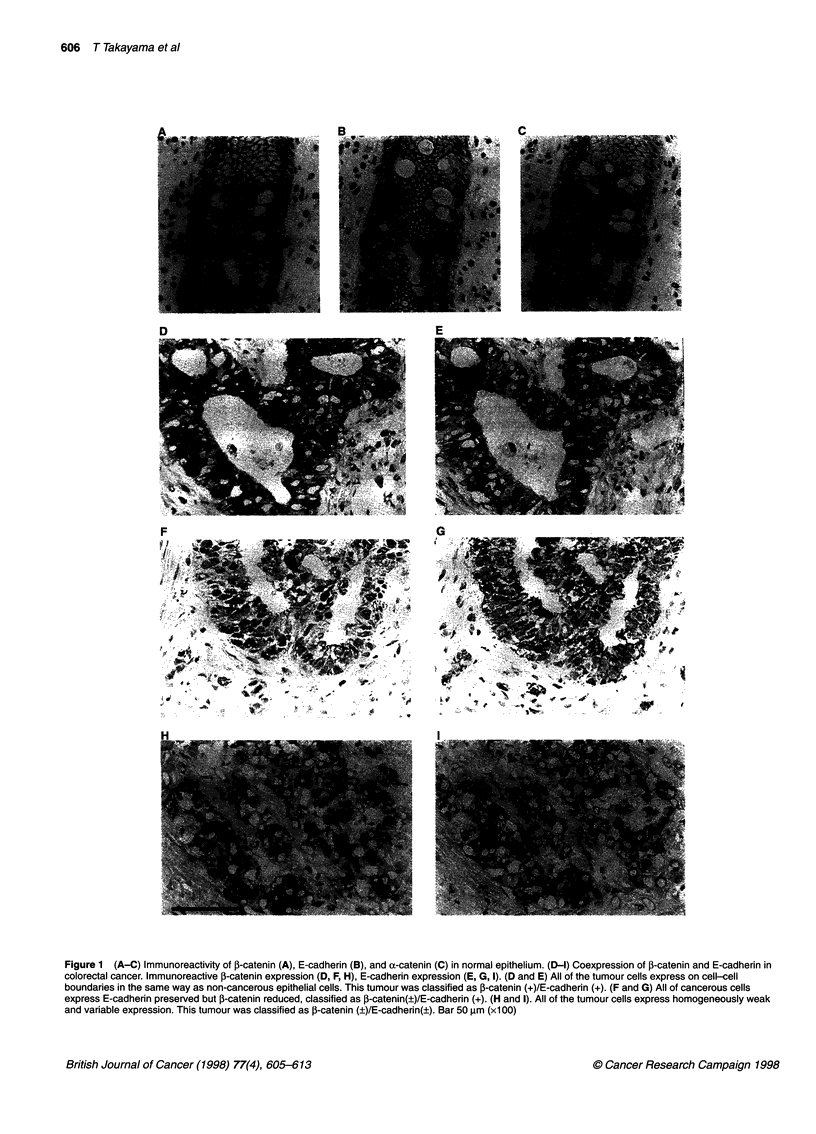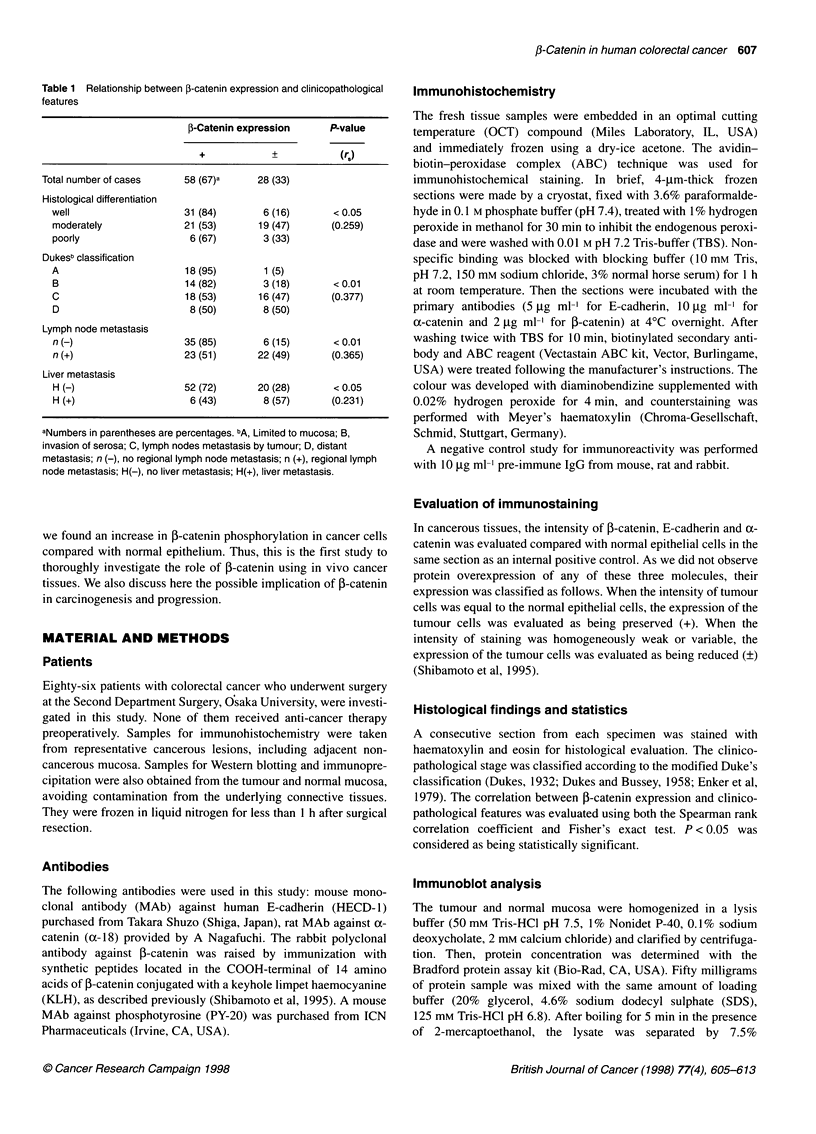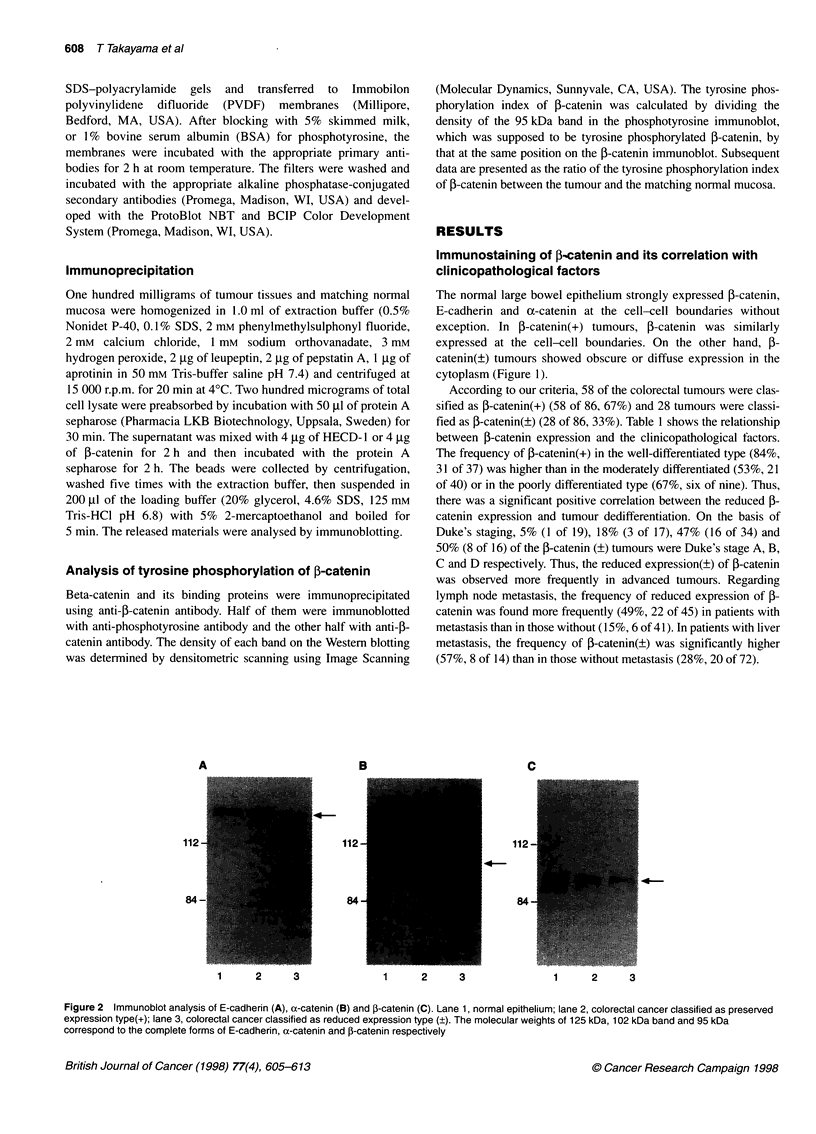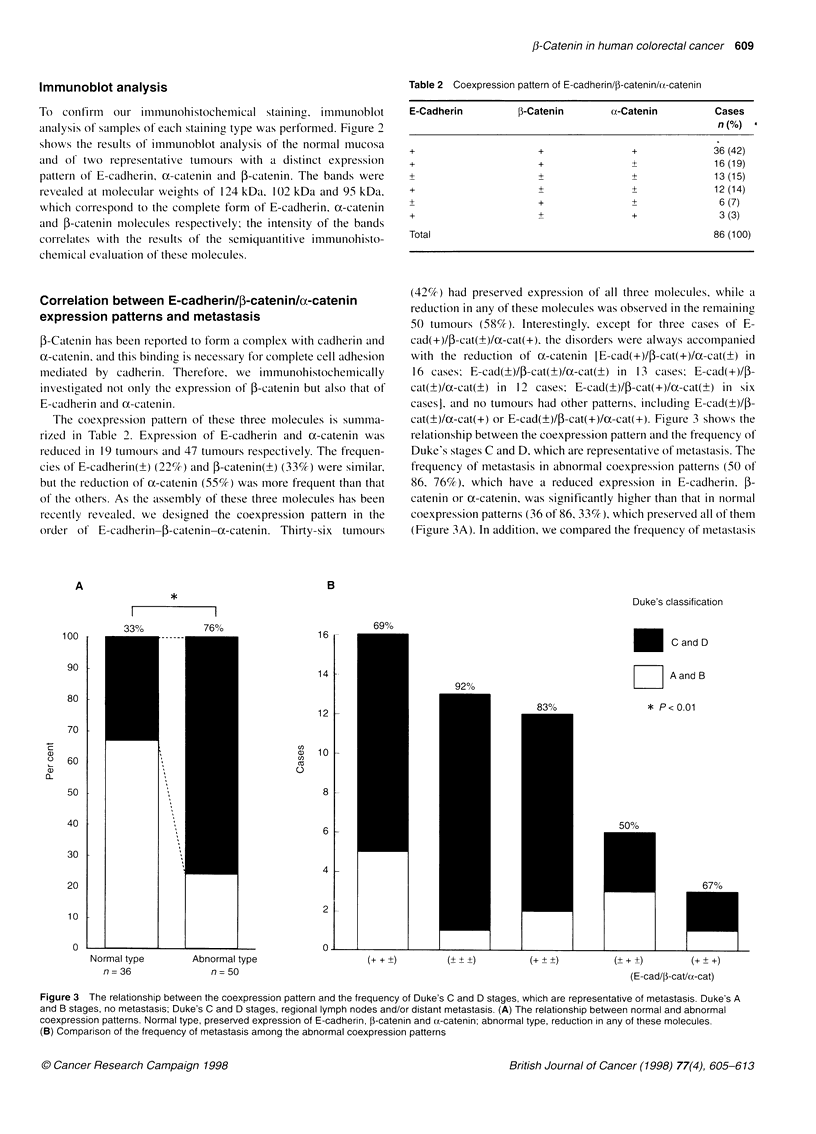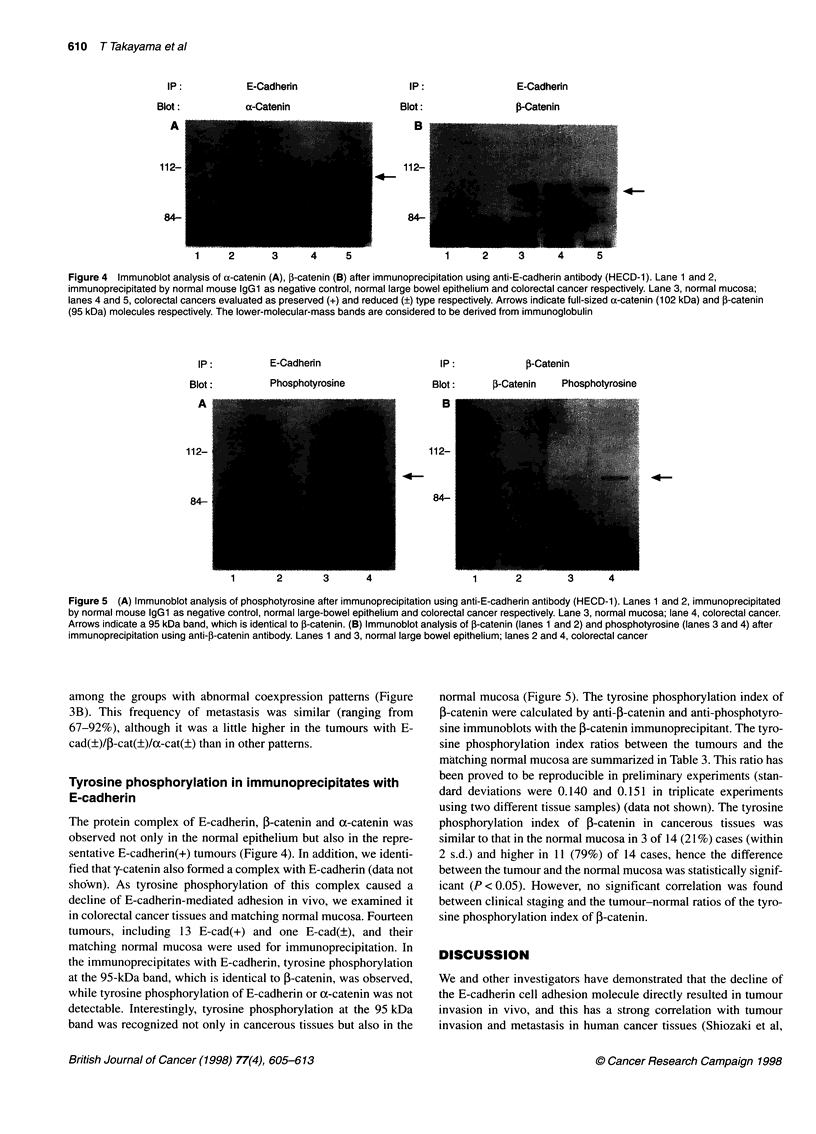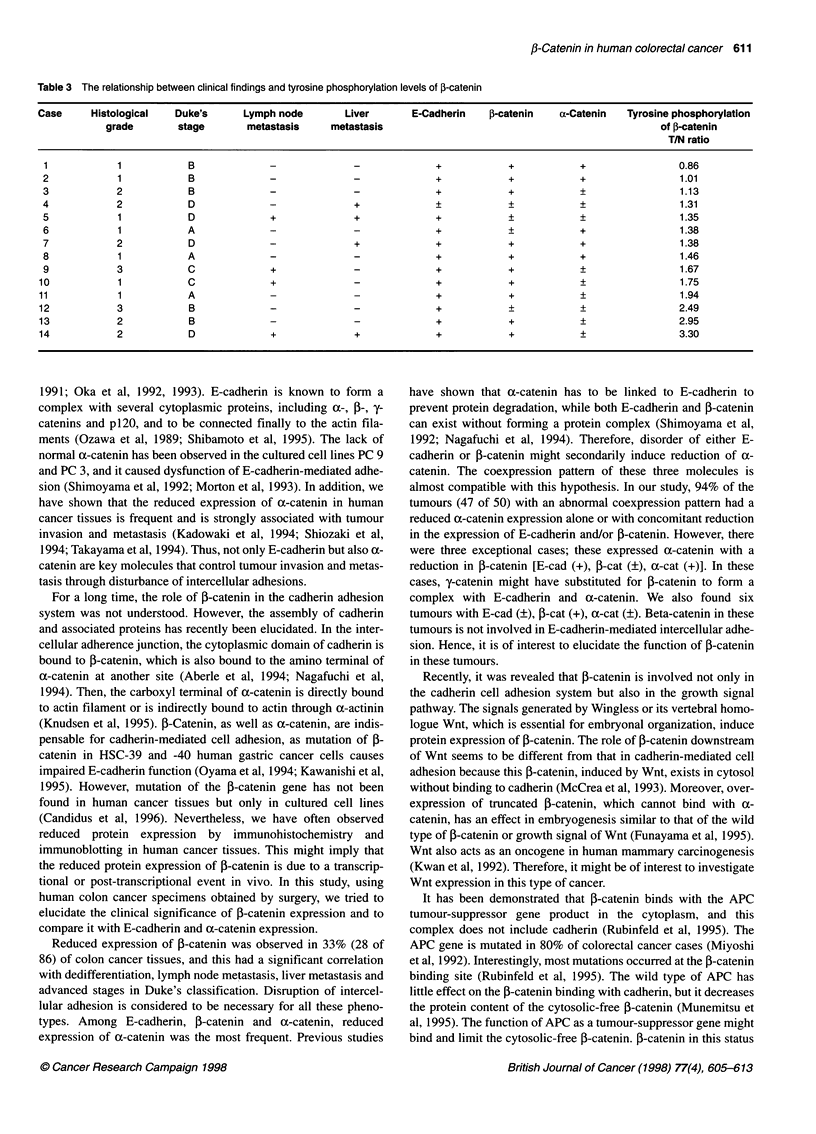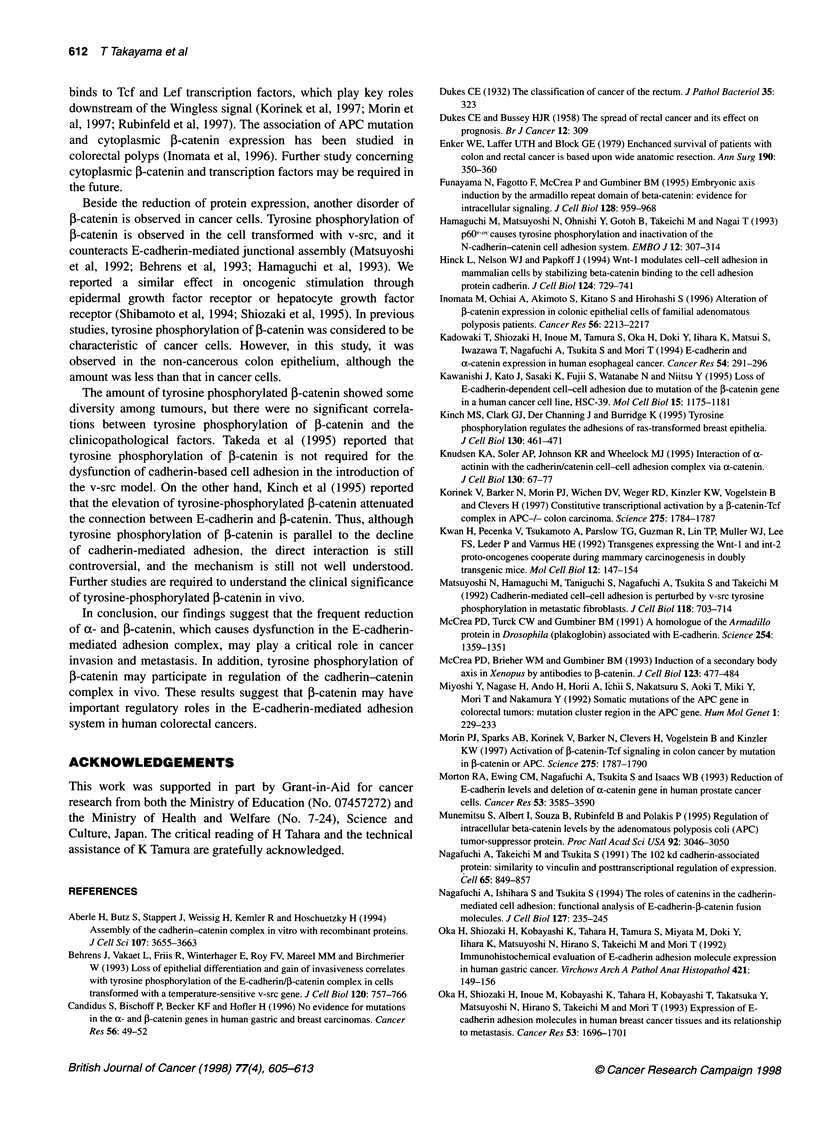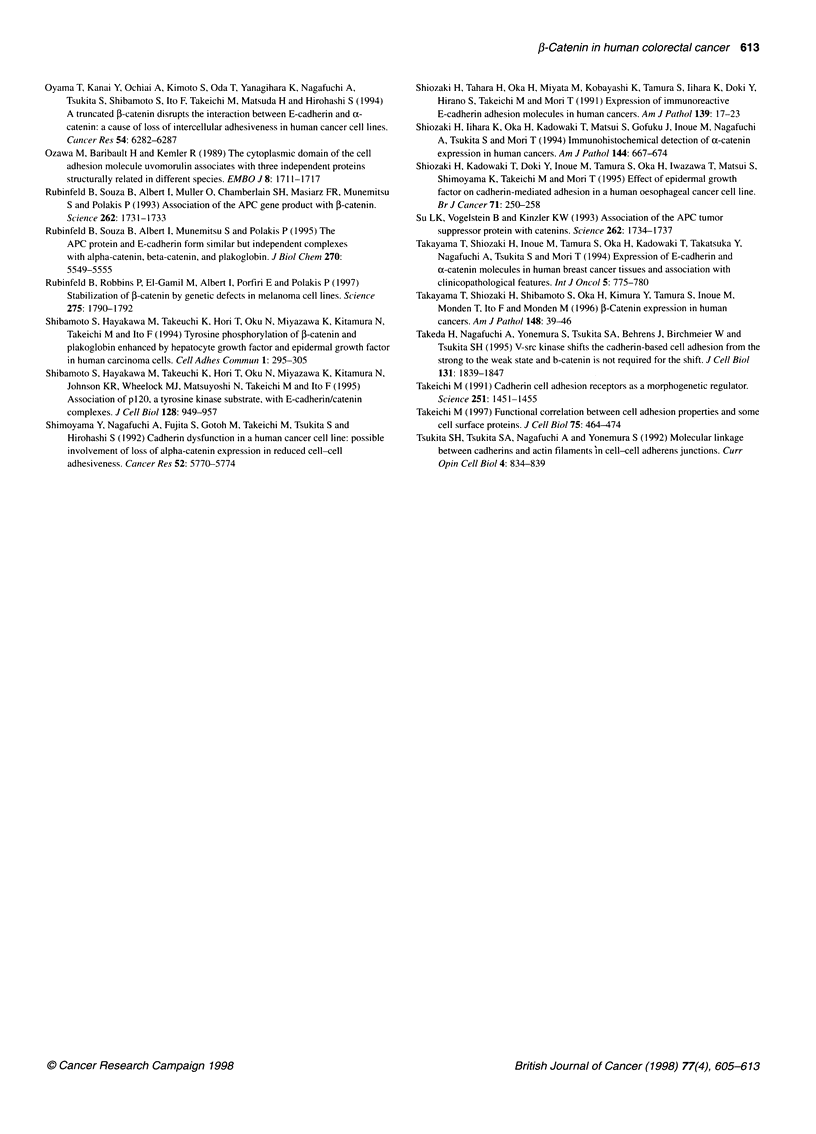# Aberrant expression and phosphorylation of beta-catenin in human colorectal cancer.

**DOI:** 10.1038/bjc.1998.97

**Published:** 1998-02

**Authors:** T. Takayama, H. Shiozaki, Y. Doki, H. Oka, M. Inoue, M. Yamamoto, S. Tamura, S. Shibamoto, F. Ito, M. Monden

**Affiliations:** Department of Surgery II, Osaka University Medical School, Suita, Japan.

## Abstract

**Images:**


British Journal of Cancer (1998) 77(4), 605–613
© 1998 Cancer Research Campaign

### Aberrant expression and phosphorylation of β-catenin in human colorectal cancer

T Takayama[1], H Shiozaki[1], Y Doki[1], H Oka[1], M Inoue[1], M Yamamoto[1], S Tamura[1], S Shibamoto[2], F Ito[2] and M Monden[1]

[1]Department of Surgery II, Osaka University Medical School, 2-2 Yamadaoka, Suita, Osaka 565, Japan; [2]Department of Biochemistry, Faculty of Pharmaceutical Sciences, Setsunan University, Hirakata, Osaka 573-01, Japan

**Summary** The cytoplasmic domain of cadherins is known to associate with the intracellular proteins, catenins, which link cadherins to the actin-based cytoskeleton. In this study, we immunohistochemically investigated the expression of β-catenin as well as E-cadherin and α-catenin in 86 human colorectal cancers, and we analysed their coexpression pattern and relationship to clinicopathological factors. In cancerous tissues, the frequency of reduced expression of β-catenin (28 of 86, 33%) was similar to that of E-cadherin (19 of 86, 22%), but less than that of α-catenin (47 of 86, 55%). All three molecules were expressed strongly, as was the normal epithelium, in 36 cases (42%), whereas the rest (50 cases, 58%) showed reduction in one of the molecules. The reduction of β-catenin expression was significantly correlated with dedifferentiation, Duke's stage, lymph node metastasis and liver metastasis. Next, we examined tyrosine phosphorylation in the protein complex immunoprecipitated with E- cadherin, as E-cadherin function is down-regulated by receptor-type tyrosine kinase in vitro. It was of interest that up-regulation of tyrosine phosphorylation of β-catenin was more frequently observed in cancerous tissues than in the matching normal mucosa.

These results suggest that β-catenin may have important regulatory roles within an E-cadherin-mediated adhesion system in human colorectal cancers.

**Keywords:** β-catenin; tyrosine phosphorylation; colorectal cancer

Cadherins are calcium dependent, homotypic cell–cell adhesion molecules that play an important role in the organization and maintenance of tissue structure (Takeichi, 1977, 1991). As detachment of cell–cell adhesion appears indispensable for cancer invasion and metastasis, this cadherin-mediated cell–cell adhesion system in cancers has been investigated. We have previously shown that E-cadherin expression in cancers is frequently impaired and is inversely correlated with the invasive behaviour of human cancers (Shiozaki et al, 1991; Oka et al, 1992, 1993).

A group of catenins, which couple the cadherins with the microfilaments of the cytoskeleton, is essential to the functions of E-cadherin (Ozawa et al, 1989; Nagafuchi et al, 1991; Tsukita et al, 1992). Deletion of the α-catenin gene, which results in decline of intercellular adhesion, has been found in PC-9 and PC-3 cancer cell lines in vitro (Shimoyama et al, 1992; Morton et al, 1993). In addition, we have reported that α-catenin is frequently decreased in cancers in vivo, suggesting that not only E-cadherin but also α-catenin play an important role in cancer invasion and metastasis (Kadowaki et al, 1994; Shiozaki et al, 1994; Takayama et al, 1994).

Another member of the catenin family, β-catenin (95 kDa), is considered to mediate interaction between E-cadherin and α-catenin, as β-catenin binds with both the cytoplasmic domain of E-cadherin and the amino terminal domain of α-catenin. However, E-cadherin cannot bind directly to α-catenin (Aberle et al, 1994). Mutated β-catenin causes impaired intercellular adhesion in HSC-39 cells, in spite of the existence of E-cadherin (Kawanishi et al, 1995). Other studies demonstrated that β-catenin was tyrosine phosphorylated in a cadherin–catenin complex and that elevation of tyrosine phosphorylation of β-catenin appears to be associated with cadherin dysfunction in vitro (Matsuyoshi et al, 1992; Behrens et al, 1993; Hamaguchi et al, 1993).

Recently, β-catenin has been proved to play a different role in embryonic morphogenesis. For example, β-catenin is involved in axis determination in Xenopus embryos, and Armadillo, a Drosophila homologue of β-catenin, is essential for establishment of segment polarity (McCrea et al, 1991). These signals may be different from cadherin-mediated cell adhesion, as they are generated by a growth factor, Wnt (or its Drosophila homologue, Wingless), and require cytosolic β-catenin not bound to E-cadherin (McCrea et al, 1993; Hinck et al, 1994). In addition, it was reported recently that the APC (adenomatous polyposis coli) tumour-suppressor gene product forms a complex with β-catenin, and disruption of this complex is a crucial step in colorectal carcinogenesis (Rubinfeld et al, 1993; Su et al, 1993). In consequence, mutation in either APC or β-catenin leads to the accumulation of cytosolic β-catenin, which binds to T-cell factor (Tcf) and lymphoid enhancer factor (Lef) transcription factors (Korinek et al, 1997; Morin et al, 1997; Rubinfeld et al, 1997)

Although we have shown that reduced expression of β-catenin has been frequently observed in human cancers, its significance in vivo is quite unknown (Takayama et al, 1996). In this study, we immunohistochemically investigated the expression of β-catenin and revealed that reduced expression of β-catenin is significantly correlated with tumour invasion and metastasis in colorectal cancer tissues. In addition, we examined the tyrosine phosphorylation of β-catenin using immunoprecipitation and Western blot, and

*Received 11 April 1997*
*Revised 3 June 1997*
*Accepted 25 June 1997*

*Correspondence to:* T Takayama

**Figure 1** (**A–C**) Immunoreactivity of β-catenin (**A**), E-cadherin (**B**), and α-catenin (**C**) in normal epithelium. (**D–I**) Coexpression of β-catenin and E-cadherin in colorectal cancer. Immunoreactive β-catenin expression (**D, F, H**), E-cadherin expression (**E, G, I**). (**D** and **E**) All of the tumour cells express on cell–cell boundaries in the same way as non-cancerous epithelial cells. This tumour was classified as β-catenin (+)/E-cadherin (+). (**F** and **G**) All of cancerous cells express E-cadherin preserved but β-catenin reduced, classified as β-catenin(±)/E-cadherin (+). (**H** and **I**). All of the tumour cells express homogeneously weak and variable expression. This tumour was classified as β-catenin (±)/E-cadherin(±). Bar 50 μm (×100)

 © Cancer Research Campaign 1998

**Table 1** Relationship between β-catenin expression and clinicopathological features

| | β-Catenin expression | | P-value |
|---|---|---|---|
| | + | ± | ($r_s$) |
| Total number of cases | 58 (67)[a] | 28 (33) | |
| Histological differentiation | | | |
| well | 31 (84) | 6 (16) | < 0.05 |
| moderately | 21 (53) | 19 (47) | (0.259) |
| poorly | 6 (67) | 3 (33) | |
| Dukes[b] classification | | | |
| A | 18 (95) | 1 (5) | |
| B | 14 (82) | 3 (18) | < 0.01 |
| C | 18 (53) | 16 (47) | (0.377) |
| D | 8 (50) | 8 (50) | |
| Lymph node metastasis | | | |
| n (–) | 35 (85) | 6 (15) | < 0.01 |
| n (+) | 23 (51) | 22 (49) | (0.365) |
| Liver metastasis | | | |
| H (–) | 52 (72) | 20 (28) | < 0.05 |
| H (+) | 6 (43) | 8 (57) | (0.231) |

[a]Numbers in parentheses are percentages. [b]A, Limited to mucosa; B, invasion of serosa; C, lymph nodes metastasis by tumour; D, distant metastasis; n (–), no regional lymph node metastasis; n (+), regional lymph node metastasis; H(–), no liver metastasis; H(+), liver metastasis.

we found an increase in β-catenin phosphorylation in cancer cells compared with normal epithelium. Thus, this is the first study to thoroughly investigate the role of β-catenin using in vivo cancer tissues. We also discuss here the possible implication of β-catenin in carcinogenesis and progression.

### MATERIAL AND METHODS

### Patients

Eighty-six patients with colorectal cancer who underwent surgery at the Second Department Surgery, Osaka University, were investigated in this study. None of them received anti-cancer therapy preoperatively. Samples for immunohistochemistry were taken from representative cancerous lesions, including adjacent non-cancerous mucosa. Samples for Western blotting and immunoprecipitation were also obtained from the tumour and normal mucosa, avoiding contamination from the underlying connective tissues. They were frozen in liquid nitrogen for less than 1 h after surgical resection.

### Antibodies

The following antibodies were used in this study: mouse monoclonal antibody (MAb) against human E-cadherin (HECD-1) purchased from Takara Shuzo (Shiga, Japan), rat MAb against α-catenin (α-18) provided by A Nagafuchi. The rabbit polyclonal antibody against β-catenin was raised by immunization with synthetic peptides located in the COOH-terminal of 14 amino acids of β-catenin conjugated with a keyhole limpet haemocyanine (KLH), as described previously (Shibamoto et al, 1995). A mouse MAb against phosphotyrosine (PY-20) was purchased from ICN Pharmaceuticals (Irvine, CA, USA).

### Immunohistochemistry

The fresh tissue samples were embedded in an optimal cutting temperature (OCT) compound (Miles Laboratory, IL, USA) and immediately frozen using a dry-ice acetone. The avidin–biotin–peroxidase complex (ABC) technique was used for immunohistochemical staining. In brief, 4-μm-thick frozen sections were made by a cryostat, fixed with 3.6% paraformaldehyde in 0.1 M phosphate buffer (pH 7.4), treated with 1% hydrogen peroxide in methanol for 30 min to inhibit the endogenous peroxidase and were washed with 0.01 M pH 7.2 Tris-buffer (TBS). Non-specific binding was blocked with blocking buffer (10 mM Tris, pH 7.2, 150 mM sodium chloride, 3% normal horse serum) for 1 h at room temperature. Then the sections were incubated with the primary antibodies (5 μg $ml^{-1}$ for E-cadherin, 10 μg $ml^{-1}$ for α-catenin and 2 μg $ml^{-1}$ for β-catenin) at 4°C overnight. After washing twice with TBS for 10 min, biotinylated secondary antibody and ABC reagent (Vectastain ABC kit, Vector, Burlingame, USA) were treated following the manufacturer's instructions. The colour was developed with diaminobendizine supplemented with 0.02% hydrogen peroxide for 4 min, and counterstaining was performed with Meyer's haematoxylin (Chroma-Gesellschaft, Schmid, Stuttgart, Germany).

A negative control study for immunoreactivity was performed with 10 μg $ml^{-1}$ pre-immune IgG from mouse, rat and rabbit.

### Evaluation of immunostaining

In cancerous tissues, the intensity of β-catenin, E-cadherin and α-catenin was evaluated compared with normal epithelial cells in the same section as an internal positive control. As we did not observe protein overexpression of any of these three molecules, their expression was classified as follows. When the intensity of tumour cells was equal to the normal epithelial cells, the expression of the tumour cells was evaluated as being preserved (+). When the intensity of staining was homogeneously weak or variable, the expression of the tumour cells was evaluated as being reduced (±) (Shibamoto et al, 1995).

### Histological findings and statistics

A consecutive section from each specimen was stained with haematoxylin and eosin for histological evaluation. The clinicopathological stage was classified according to the modified Duke's classification (Dukes, 1932; Dukes and Bussey, 1958; Enker et al, 1979). The correlation between β-catenin expression and clinicopathological features was evaluated using both the Spearman rank correlation coefficient and Fisher's exact test. $P < 0.05$ was considered as being statistically significant.

### Immunoblot analysis

The tumour and normal mucosa were homogenized in a lysis buffer (50 mM Tris-HCl pH 7.5, 1% Nonidet P-40, 0.1% sodium deoxycholate, 2 mM calcium chloride) and clarified by centrifugation. Then, protein concentration was determined with the Bradford protein assay kit (Bio-Rad, CA, USA). Fifty milligrams of protein sample was mixed with the same amount of loading buffer (20% glycerol, 4.6% sodium dodecyl sulphate (SDS), 125 mM Tris-HCl pH 6.8). After boiling for 5 min in the presence of 2-mercaptoethanol, the lysate was separated by 7.5%

© Cancer Research Campaign 1998 

SDS–polyacrylamide gels and transferred to Immobilon polyvinylidene difluoride (PVDF) membranes (Millipore, Bedford, MA, USA). After blocking with 5% skimmed milk, or 1% bovine serum albumin (BSA) for phosphotyrosine, the membranes were incubated with the appropriate primary antibodies for 2 h at room temperature. The filters were washed and incubated with the appropriate alkaline phosphatase-conjugated secondary antibodies (Promega, Madison, WI, USA) and developed with the ProtoBlot NBT and BCIP Color Development System (Promega, Madison, WI, USA).

### Immunoprecipitation

One hundred milligrams of tumour tissues and matching normal mucosa were homogenized in 1.0 ml of extraction buffer (0.5% Nonidet P-40, 0.1% SDS, 2 mM phenylmethylsulphonyl fluoride, 2 mM calcium chloride, 1 mM sodium orthovanadate, 3 mM hydrogen peroxide, 2 μg of leupeptin, 2 μg of pepstatin A, 1 μg of aprotinin in 50 mM Tris-buffer saline pH 7.4) and centrifuged at 15 000 r.p.m. for 20 min at 4°C. Two hundred micrograms of total cell lysate were preabsorbed by incubation with 50 μl of protein A sepharose (Pharmacia LKB Biotechnology, Uppsala, Sweden) for 30 min. The supernatant was mixed with 4 μg of HECD-1 or 4 μg of β-catenin for 2 h and then incubated with the protein A sepharose for 2 h. The beads were collected by centrifugation, washed five times with the extraction buffer, then suspended in 200 μl of the loading buffer (20% glycerol, 4.6% SDS, 125 mM Tris-HCl pH 6.8) with 5% 2-mercaptoethanol and boiled for 5 min. The released materials were analysed by immunoblotting.

### Analysis of tyrosine phosphorylation of β-catenin

Beta-catenin and its binding proteins were immunoprecipitated using anti-β-catenin antibody. Half of them were immunoblotted with anti-phosphotyrosine antibody and the other half with anti-β-catenin antibody. The density of each band on the Western blotting was determined by densitometric scanning using Image Scanning (Molecular Dynamics, Sunnyvale, CA, USA). The tyrosine phosphorylation index of β-catenin was calculated by dividing the density of the 95 kDa band in the phosphotyrosine immunoblot, which was supposed to be tyrosine phosphorylated β-catenin, by that at the same position on the β-catenin immunoblot. Subsequent data are presented as the ratio of the tyrosine phosphorylation index of β-catenin between the tumour and the matching normal mucosa.

### RESULTS

### Immunostaining of β-catenin and its correlation with clinicopathological factors

The normal large bowel epithelium strongly expressed β-catenin, E-cadherin and α-catenin at the cell–cell boundaries without exception. In β-catenin(+) tumours, β-catenin was similarly expressed at the cell–cell boundaries. On the other hand, β-catenin(±) tumours showed obscure or diffuse expression in the cytoplasm (Figure 1).

According to our criteria, 58 of the colorectal tumours were classified as β-catenin(+) (58 of 86, 67%) and 28 tumours were classified as β-catenin(±) (28 of 86, 33%). Table 1 shows the relationship between β-catenin expression and the clinicopathological factors. The frequency of β-catenin(+) in the well-differentiated type (84%, 31 of 37) was higher than in the moderately differentiated (53%, 21 of 40) or in the poorly differentiated type (67%, six of nine). Thus, there was a significant positive correlation between the reduced β-catenin expression and tumour dedifferentiation. On the basis of Duke's staging, 5% (1 of 19), 18% (3 of 17), 47% (16 of 34) and 50% (8 of 16) of the β-catenin (±) tumours were Duke's stage A, B, C and D respectively. Thus, the reduced expression(±) of β-catenin was observed more frequently in advanced tumours. Regarding lymph node metastasis, the frequency of reduced expression of β-catenin was found more frequently (49%, 22 of 45) in patients with metastasis than in those without (15%, 6 of 41). In patients with liver metastasis, the frequency of β-catenin(±) was significantly higher (57%, 8 of 14) than in those without metastasis (28%, 20 of 72).

**Figure 2** Immunoblot analysis of E-cadherin (**A**), α-catenin (**B**) and β-catenin (**C**). Lane 1, normal epithelium; lane 2, colorectal cancer classified as preserved expression type(+); lane 3, colorectal cancer classified as reduced expression type (±). The molecular weights of 125 kDa, 102 kDa band and 95 kDa correspond to the complete forms of E-cadherin, α-catenin and β-catenin respectively

© Cancer Research Campaign 1998

### Immunoblot analysis

To confirm our immunohistochemical staining, immunoblot analysis of samples of each staining type was performed. Figure 2 shows the results of immunoblot analysis of the normal mucosa and of two representative tumours with a distinct expression pattern of E-cadherin, α-catenin and β-catenin. The bands were revealed at molecular weights of 124 kDa, 102 kDa and 95 kDa, which correspond to the complete form of E-cadherin, α-catenin and β-catenin molecules respectively; the intensity of the bands correlates with the results of the semiquantitive immunohistochemical evaluation of these molecules.

### Correlation between E-cadherin/β-catenin/α-catenin expression patterns and metastasis

β-Catenin has been reported to form a complex with cadherin and α-catenin, and this binding is necessary for complete cell adhesion mediated by cadherin. Therefore, we immunohistochemically investigated not only the expression of β-catenin but also that of E-cadherin and α-catenin.

The coexpression pattern of these three molecules is summarized in Table 2. Expression of E-cadherin and α-catenin was reduced in 19 tumours and 47 tumours respectively. The frequencies of E-cadherin(±) (22%) and β-catenin(±) (33%) were similar, but the reduction of α-catenin (55%) was more frequent than that of the others. As the assembly of these three molecules has been recently revealed, we designed the coexpression pattern in the order of E-cadherin–β-catenin–α-catenin. Thirty-six tumours (42%) had preserved expression of all three molecules, while a reduction in any of these molecules was observed in the remaining 50 tumours (58%). Interestingly, except for three cases of E-cad(+)/β-cat(±)/α-cat(+), the disorders were always accompanied with the reduction of α-catenin [E-cad(+)/β-cat(+)/α-cat(±) in 16 cases; E-cad(±)/β-cat(±)/α-cat(±) in 13 cases; E-cad(+)/β-cat(±)/α-cat(±) in 12 cases; E-cad(±)/β-cat(+)/α-cat(±) in six cases], and no tumours had other patterns, including E-cad(±)/β-cat(±)/α-cat(+) or E-cad(±)/β-cat(+)/α-cat(+). Figure 3 shows the relationship between the coexpression pattern and the frequency of Duke's stages C and D, which are representative of metastasis. The frequency of metastasis in abnormal coexpression patterns (50 of 86, 76%), which have a reduced expression in E-cadherin, β-catenin or α-catenin, was significantly higher than that in normal coexpression patterns (36 of 86, 33%), which preserved all of them (Figure 3A). In addition, we compared the frequency of metastasis

**Table 2** Coexpression pattern of E-cadherin/β-catenin/α-catenin

| E-Cadherin | β-Catenin | α-Catenin | Cases n (%) |
|---|---|---|---|
| + | + | + | 36 (42) |
| + | + | ± | 16 (19) |
| ± | ± | ± | 13 (15) |
| + | ± | ± | 12 (14) |
| ± | + | ± | 6 (7) |
| + | ± | + | 3 (3) |
| Total | | | 86 (100) |

**Figure 3** The relationship between the coexpression pattern and the frequency of Duke's C and D stages, which are representative of metastasis. Duke's A and B stages, no metastasis; Duke's C and D stages, regional lymph nodes and/or distant metastasis. (**A**) The relationship between normal and abnormal coexpression patterns. Normal type, preserved expression of E-cadherin, β-catenin and α-catenin; abnormal type, reduction in any of these molecules. (**B**) Comparison of the frequency of metastasis among the abnormal coexpression patterns

**Figure 4** Immunoblot analysis of α-catenin (**A**), β-catenin (**B**) after immunoprecipitation using anti-E-cadherin antibody (HECD-1). Lane 1 and 2, immunoprecipitated by normal mouse IgG1 as negative control, normal large bowel epithelium and colorectal cancer respectively. Lane 3, normal mucosa; lanes 4 and 5, colorectal cancers evaluated as preserved (+) and reduced (±) type respectively. Arrows indicate full-sized α-catenin (102 kDa) and β-catenin (95 kDa) molecules respectively. The lower-molecular-mass bands are considered to be derived from immunoglobulin

**Figure 5** (**A**) Immunoblot analysis of phosphotyrosine after immunoprecipitation using anti-E-cadherin antibody (HECD-1). Lanes 1 and 2, immunoprecipitated by normal mouse IgG1 as negative control, normal large-bowel epithelium and colorectal cancer respectively. Lane 3, normal mucosa; lane 4, colorectal cancer. Arrows indicate a 95 kDa band, which is identical to β-catenin. (**B**) Immunoblot analysis of β-catenin (lanes 1 and 2) and phosphotyrosine (lanes 3 and 4) after immunoprecipitation using anti-β-catenin antibody. Lanes 1 and 3, normal large bowel epithelium; lanes 2 and 4, colorectal cancer among the groups with abnormal coexpression patterns (Figure 3B). This frequency of metastasis was similar (ranging from 67–92%), although it was a little higher in the tumours with E-cad(±)/β-cat(±)/α-cat(±) than in other patterns.

### Tyrosine phosphorylation in immunoprecipitates with E-cadherin

The protein complex of E-cadherin, β-catenin and α-catenin was observed not only in the normal epithelium but also in the representative E-cadherin(+) tumours (Figure 4). In addition, we identified that γ-catenin also formed a complex with E-cadherin (data not shown). As tyrosine phosphorylation of this complex caused a decline of E-cadherin-mediated adhesion in vivo, we examined it in colorectal cancer tissues and matching normal mucosa. Fourteen tumours, including 13 E-cad(+) and one E-cad(±), and their matching normal mucosa were used for immunoprecipitation. In the immunoprecipitates with E-cadherin, tyrosine phosphorylation at the 95-kDa band, which is identical to β-catenin, was observed, while tyrosine phosphorylation of E-cadherin or α-catenin was not detectable. Interestingly, tyrosine phosphorylation at the 95 kDa band was recognized not only in cancerous tissues but also in the normal mucosa (Figure 5). The tyrosine phosphorylation index of β-catenin were calculated by anti-β-catenin and anti-phosphotyrosine immunoblots with the β-catenin immunoprecipitant. The tyrosine phosphorylation index ratios between the tumours and the matching normal mucosa are summarized in Table 3. This ratio has been proved to be reproducible in preliminary experiments (standard deviations were 0.140 and 0.151 in triplicate experiments using two different tissue samples) (data not shown). The tyrosine phosphorylation index of β-catenin in cancerous tissues was similar to that in the normal mucosa in 3 of 14 (21%) cases (within 2 s.d.) and higher in 11 (79%) of 14 cases, hence the difference between the tumour and the normal mucosa was statistically significant ($P < 0.05$). However, no significant correlation was found between clinical staging and the tumour–normal ratios of the tyrosine phosphorylation index of β-catenin.

### DISCUSSION

We and other investigators have demonstrated that the decline of the E-cadherin cell adhesion molecule directly resulted in tumour invasion in vivo, and this has a strong correlation with tumour invasion and metastasis in human cancer tissues (Shiozaki et al,

 © Cancer Research Campaign 1998

Table 3 The relationship between clinical findings and tyrosine phosphorylation levels of β-catenin

| Case | Histological grade | Duke's stage | Lymph node metastasis | Liver metastasis | E-Cadherin | β-catenin | α-Catenin | Tyrosine phosphorylation of β-catenin T/N ratio |
|---|---|---|---|---|---|---|---|---|
| 1 | 1 | B | – | – | + | + | + | 0.86 |
| 2 | 1 | B | – | – | + | + | + | 1.01 |
| 3 | 2 | B | – | – | + | + | ± | 1.13 |
| 4 | 2 | D | – | + | ± | ± | ± | 1.31 |
| 5 | 1 | D | + | + | + | ± | ± | 1.35 |
| 6 | 1 | A | – | – | + | ± | + | 1.38 |
| 7 | 2 | D | – | + | + | + | + | 1.38 |
| 8 | 1 | A | – | – | + | + | + | 1.46 |
| 9 | 3 | C | + | – | + | + | ± | 1.67 |
| 10 | 1 | C | + | – | + | + | ± | 1.75 |
| 11 | 1 | A | – | – | + | + | ± | 1.94 |
| 12 | 3 | B | – | – | + | ± | ± | 2.49 |
| 13 | 2 | B | – | – | + | + | ± | 2.95 |
| 14 | 2 | D | + | + | + | + | ± | 3.30 |

1991; Oka et al, 1992, 1993). E-cadherin is known to form a complex with several cytoplasmic proteins, including α-, β-, γ-catenins and p120, and to be connected finally to the actin filaments (Ozawa et al, 1989; Shibamoto et al, 1995). The lack of normal α-catenin has been observed in the cultured cell lines PC 9 and PC 3, and it caused dysfunction of E-cadherin-mediated adhesion (Shimoyama et al, 1992; Morton et al, 1993). In addition, we have shown that the reduced expression of α-catenin in human cancer tissues is frequent and is strongly associated with tumour invasion and metastasis (Kadowaki et al, 1994; Shiozaki et al, 1994; Takayama et al, 1994). Thus, not only E-cadherin but also α-catenin are key molecules that control tumour invasion and metastasis through disturbance of intercellular adhesions.

For a long time, the role of β-catenin in the cadherin adhesion system was not understood. However, the assembly of cadherin and associated proteins has recently been elucidated. In the intercellular adherence junction, the cytoplasmic domain of cadherin is bound to β-catenin, which is also bound to the amino terminal of α-catenin at another site (Aberle et al, 1994; Nagafuchi et al, 1994). Then, the carboxyl terminal of α-catenin is directly bound to actin filament or is indirectly bound to actin through α-actinin (Knudsen et al, 1995). β-Catenin, as well as α-catenin, are indispensable for cadherin-mediated cell adhesion, as mutation of β-catenin in HSC-39 and -40 human gastric cancer cells causes impaired E-cadherin function (Oyama et al, 1994; Kawanishi et al, 1995). However, mutation of the β-catenin gene has not been found in human cancer tissues but only in cultured cell lines (Candidus et al, 1996). Nevertheless, we have often observed reduced protein expression by immunohistochemistry and immunoblotting in human cancer tissues. This might imply that the reduced protein expression of β-catenin is due to a transcriptional or post-transcriptional event in vivo. In this study, using human colon cancer specimens obtained by surgery, we tried to elucidate the clinical significance of β-catenin expression and to compare it with E-cadherin and α-catenin expression.

Reduced expression of β-catenin was observed in 33% (28 of 86) of colon cancer tissues, and this had a significant correlation with dedifferentiation, lymph node metastasis, liver metastasis and advanced stages in Duke's classification. Disruption of intercellular adhesion is considered to be necessary for all these phenotypes. Among E-cadherin, β-catenin and α-catenin, reduced expression of α-catenin was the most frequent. Previous studies have shown that α-catenin has to be linked to E-cadherin to prevent protein degradation, while both E-cadherin and β-catenin can exist without forming a protein complex (Shimoyama et al, 1992; Nagafuchi et al, 1994). Therefore, disorder of either E-cadherin or β-catenin might secondarily induce reduction of α-catenin. The coexpression pattern of these three molecules is almost compatible with this hypothesis. In our study, 94% of the tumours (47 of 50) with an abnormal coexpression pattern had a reduced α-catenin expression alone or with concomitant reduction in the expression of E-cadherin and/or β-catenin. However, there were three exceptional cases; these expressed α-catenin with a reduction in β-catenin [E-cad (+), β-cat (±), α-cat (+)]. In these cases, γ-catenin might have substituted for β-catenin to form a complex with E-cadherin and α-catenin. We also found six tumours with E-cad (±), β-cat (+), α-cat (±). Beta-catenin in these tumours is not involved in E-cadherin-mediated intercellular adhesion. Hence, it is of interest to elucidate the function of β-catenin in these tumours.

Recently, it was revealed that β-catenin is involved not only in the cadherin cell adhesion system but also in the growth signal pathway. The signals generated by Wingless or its vertebral homologue Wnt, which is essential for embryonal organization, induce protein expression of β-catenin. The role of β-catenin downstream of Wnt seems to be different from that in cadherin-mediated cell adhesion because this β-catenin, induced by Wnt, exists in cytosol without binding to cadherin (McCrea et al, 1993). Moreover, overexpression of truncated β-catenin, which cannot bind with α-catenin, has an effect in embryogenesis similar to that of the wild type of β-catenin or growth signal of Wnt (Funayama et al, 1995). Wnt also acts as an oncogene in human mammary carcinogenesis (Kwan et al, 1992). Therefore, it might be of interest to investigate Wnt expression in this type of cancer.

It has been demonstrated that β-catenin binds with the APC tumour-suppressor gene product in the cytoplasm, and this complex does not include cadherin (Rubinfeld et al, 1995). The APC gene is mutated in 80% of colorectal cancer cases (Miyoshi et al, 1992). Interestingly, most mutations occurred at the β-catenin binding site (Rubinfeld et al, 1995). The wild type of APC has little effect on the β-catenin binding with cadherin, but it decreases the protein content of the cytosolic-free β-catenin (Munemitsu et al, 1995). The function of APC as a tumour-suppressor gene might bind and limit the cytosolic-free β-catenin. β-catenin in this status

© Cancer Research Campaign 1998 

binds to Tcf and Lef transcription factors, which play key roles downstream of the Wingless signal (Korinek et al, 1997; Morin et al, 1997; Rubinfeld et al, 1997). The association of APC mutation and cytoplasmic β-catenin expression has been studied in colorectal polyps (Inomata et al, 1996). Further study concerning cytoplasmic β-catenin and transcription factors may be required in the future.

Beside the reduction of protein expression, another disorder of β-catenin is observed in cancer cells. Tyrosine phosphorylation of β-catenin is observed in the cell transformed with v-src, and it counteracts E-cadherin-mediated junctional assembly (Matsuyoshi et al, 1992; Behrens et al, 1993; Hamaguchi et al, 1993). We reported a similar effect in oncogenic stimulation through epidermal growth factor receptor or hepatocyte growth factor receptor (Shibamoto et al, 1994; Shiozaki et al, 1995). In previous studies, tyrosine phosphorylation of β-catenin was considered to be characteristic of cancer cells. However, in this study, it was observed in the non-cancerous colon epithelium, although the amount was less than that in cancer cells.

The amount of tyrosine phosphorylated β-catenin showed some diversity among tumours, but there were no significant correlations between tyrosine phosphorylation of β-catenin and the clinicopathological factors. Takeda et al (1995) reported that tyrosine phosphorylation of β-catenin is not required for the dysfunction of cadherin-based cell adhesion in the introduction of the v-src model. On the other hand, Kinch et al (1995) reported that the elevation of tyrosine-phosphorylated β-catenin attenuated the connection between E-cadherin and β-catenin. Thus, although tyrosine phosphorylation of β-catenin is parallel to the decline of cadherin-mediated adhesion, the direct interaction is still controversial, and the mechanism is still not well understood. Further studies are required to understand the clinical significance of tyrosine-phosphorylated β-catenin in vivo.

In conclusion, our findings suggest that the frequent reduction of α- and β-catenin, which causes dysfunction in the E-cadherin-mediated adhesion complex, may play a critical role in cancer invasion and metastasis. In addition, tyrosine phosphorylation of β-catenin may participate in regulation of the cadherin–catenin complex in vivo. These results suggest that β-catenin may have important regulatory roles in the E-cadherin-mediated adhesion system in human colorectal cancers.

### ACKNOWLEDGEMENTS

This work was supported in part by Grant-in-Aid for cancer research from both the Ministry of Education (No. 07457272) and the Ministry of Health and Welfare (No. 7-24), Science and Culture, Japan. The critical reading of H Tahara and the technical assistance of K Tamura are gratefully acknowledged.

### REFERENCES

Aberle H, Butz S, Stappert J, Weissig H, Kemler R and Hoschuetzky H (1994) Assembly of the cadherin–catenin complex in vitro with recombinant proteins. *J Cell Sci* **107**: 3655–3663

Behrens J, Vakaet L, Friis R, Winterhager E, Roy FV, Mareel MM and Birchmerier W (1993) Loss of epithelial differentiation and gain of invasiveness correlates with tyrosine phosphorylation of the E-cadherin/β-catenin complex in cells transformed with a temperature-sensitive v-src gene. *J Cell Biol* **120**: 757–766

Candidus S, Bischoff P, Becker KF and Hofler H (1996) No evidence for mutations in the α- and β-catenin genes in human gastric and breast carcinomas. *Cancer Res* **56**: 49–52

Dukes CE (1932) The classification of cancer of the rectum. *J Pathol Bacteriol* **35**: 323

Dukes CE and Bussey HJR (1958) The spread of rectal cancer and its effect on prognosis. *Br J Cancer* **12**: 309

Enker WE, Laffer UTH and Block GE (1979) Enchanced survival of patients with colon and rectal cancer is based upon wide anatomic resection. *Ann Surg* **190**: 350–360

Funayama N, Fagotto F, McCrea P and Gumbiner BM (1995) Embryonic axis induction by the armadillo repeat domain of beta-catenin: evidence for intracellular signaling. *J Cell Biol* **128**: 959–968

Hamaguchi M, Matsuyoshi N, Ohnishi Y, Gotoh B, Takeichi M and Nagai T (1993) $p60^{v-src}$ causes tyrosine phosphorylation and inactivation of the N-cadherin–catenin cell adhesion system. *EMBO J* **12**: 307–314

Hinck L, Nelson WJ and Papkoff J (1994) Wnt-1 modulates cell–cell adhesion in mammalian cells by stabilizing beta-catenin binding to the cell adhesion protein cadherin. *J Cell Biol* **124**: 729–741

Inomata M, Ochiai A, Akimoto S, Kitano S and Hirohashi S (1996) Alteration of β-catenin expression in colonic epithelial cells of familial adenomatous polyposis patients. *Cancer Res* **56**: 2213–2217

Kadowaki T, Shiozaki H, Inoue M, Tamura S, Oka H, Doki Y, Iihara K, Matsui S, Iwazawa T, Nagafuchi A, Tsukita S and Mori T (1994) E-cadherin and α-catenin expression in human esophageal cancer. *Cancer Res* **54**: 291–296

Kawanishi J, Kato J, Sasaki K, Fujii S, Watanabe N and Niitsu Y (1995) Loss of E-cadherin-dependent cell–cell adhesion due to mutation of the β-catenin gene in a human cancer cell line, HSC-39. *Mol Cell Biol* **15**: 1175–1181

Kinch MS, Clark GJ, Der Channing J and Burridge K (1995) Tyrosine phosphorylation regulates the adhesions of ras-transformed breast epithelia. *J Cell Biol* **130**: 461–471

Knudsen KA, Soler AP, Johnson KR and Wheelock MJ (1995) Interaction of α-actinin with the cadherin/catenin cell–cell adhesion complex via α-catenin. *J Cell Biol* **130**: 67–77

Korinek V, Barker N, Morin PJ, Wichen DV, Weger RD, Kinzler KW, Vogelstein B and Clevers H (1997) Constitutive transcriptional activation by a β-catenin-Tcf complex in APC–/– colon carcinoma. *Science* **275**: 1784–1787

Kwan H, Pecenka V, Tsukamoto A, Parslow TG, Guzman R, Lin TP, Muller WJ, Lee FS, Leder P and Varmus HE (1992) Transgenes expressing the Wnt-1 and int-2 proto-oncogenes cooperate during mammary carcinogenesis in doubly transgenic mice. *Mol Cell Biol* **12**: 147–154

Matsuyoshi N, Hamaguchi M, Taniguchi S, Nagafuchi A, Tsukita S and Takeichi M (1992) Cadherin-mediated cell–cell adhesion is perturbed by v-src tyrosine phosphorylation in metastatic fibroblasts. *J Cell Biol* **118**: 703–714

McCrea PD, Turck CW and Gumbiner BM (1991) A homologue of the *Armadillo* protein in *Drosophila* (plakoglobin) associated with E-cadherin. *Science* **254**: 1359–1351

McCrea PD, Brieher WM and Gumbiner BM (1993) Induction of a secondary body axis in *Xenopus* by antibodies to β-catenin. *J Cell Biol* **123**: 477–484

Miyoshi Y, Nagase H, Ando H, Horii A, Ichii S, Nakatsuru S, Aoki T, Miki Y, Mori T and Nakamura Y (1992) Somatic mutations of the APC gene in colorectal tumors: mutation cluster region in the APC gene. *Hum Mol Genet* **1**: 229–233

Morin PJ, Sparks AB, Korinek V, Barker N, Clevers H, Vogelstein B and Kinzler KW (1997) Activation of β-catenin-Tcf signaling in colon cancer by mutation in β-catenin or APC. *Science* **275**: 1787–1790

Morton RA, Ewing CM, Nagafuchi A, Tsukita S and Isaacs WB (1993) Reduction of E-cadherin levels and deletion of α-catenin gene in human prostate cancer cells. *Cancer Res* **53**: 3585–3590

Munemitsu S, Albert I, Souza B, Rubinfeld B and Polakis P (1995) Regulation of intracellular beta-catenin levels by the adenomatous polyposis coli (APC) tumor-suppressor protein. *Proc Natl Acad Sci USA* **92**: 3046–3050

Nagafuchi A, Takeichi M and Tsukita S (1991) The 102 kd cadherin-associated protein: similarity to vinculin and posttranscriptional regulation of expression. *Cell* **65**: 849–857

Nagafuchi A, Ishihara S and Tsukita S (1994) The roles of catenins in the cadherin-mediated cell adhesion: functional analysis of E-cadherin-β-catenin fusion molecules. *J Cell Biol* **127**: 235–245

Oka H, Shiozaki H, Kobayashi K, Tahara H, Tamura S, Miyata M, Doki Y, Iihara K, Matsuyoshi N, Hirano S, Takeichi M and Mori T (1992) Immunohistochemical evaluation of E-cadherin adhesion molecule expression in human gastric cancer. *Virchows Arch A Pathol Anat Histopathol* **421**: 149–156

Oka H, Shiozaki H, Inoue M, Kobayashi K, Tahara H, Kobayashi T, Takatsuka Y, Matsuyoshi N, Hirano S, Takeichi M and Mori T (1993) Expression of E-cadherin adhesion molecules in human breast cancer tissues and its relationship to metastasis. *Cancer Res* **53**: 1696–1701

 © Cancer Research Campaign 1998

Oyama T, Kanai Y, Ochiai A, Kimoto S, Oda T, Yanagihara K, Nagafuchi A, Tsukita S, Shibamoto S, Ito F, Takeichi M, Matsuda H and Hirohashi S (1994) A truncated β-catenin disrupts the interaction between E-cadherin and α-catenin: a cause of loss of intercellular adhesiveness in human cancer cell lines. *Cancer Res* **54**: 6282–6287

Ozawa M, Baribault H and Kemler R (1989) The cytoplasmic domain of the cell adhesion molecule uvomorulin associates with three independent proteins structurally related in different species. *EMBO J* **8**: 1711–1717

Rubinfeld B, Souza B, Albert I, Muller O, Chamberlain SH, Masiarz FR, Munemitsu S and Polakis P (1993) Association of the APC gene product with β-catenin. *Science* **262**: 1731–1733

Rubinfeld B, Souza B, Albert I, Munemitsu S and Polakis P (1995) The APC protein and E-cadherin form similar but independent complexes with alpha-catenin, beta-catenin, and plakoglobin. *J Biol Chem* **270**: 5549–5555

Rubinfeld B, Robbins P, El-Gamil M, Albert I, Porfiri E and Polakis P (1997) Stabilization of β-catenin by genetic defects in melanoma cell lines. *Science* **275**: 1790–1792

Shibamoto S, Hayakawa M, Takeuchi K, Hori T, Oku N, Miyazawa K, Kitamura N, Takeichi M and Ito F (1994) Tyrosine phosphorylation of β-catenin and plakoglobin enhanced by hepatocyte growth factor and epidermal growth factor in human carcinoma cells. *Cell Adhes Commun* **1**: 295–305

Shibamoto S, Hayakawa M, Takeuchi K, Hori T, Oku N, Miyazawa K, Kitamura N, Johnson KR, Wheelock MJ, Matsuyoshi N, Takeichi M and Ito F (1995) Association of p120, a tyrosine kinase substrate, with E-cadherin/catenin complexes. *J Cell Biol* **128**: 949–957

Shimoyama Y, Nagafuchi A, Fujita S, Gotoh M, Takeichi M, Tsukita S and Hirohashi S (1992) Cadherin dysfunction in a human cancer cell line: possible involvement of loss of alpha-catenin expression in reduced cell–cell adhesiveness. *Cancer Res* **52**: 5770–5774

Shiozaki H, Tahara H, Oka H, Miyata M, Kobayashi K, Tamura S, Iihara K, Doki Y, Hirano S, Takeichi M and Mori T (1991) Expression of immunoreactive E-cadherin adhesion molecules in human cancers. *Am J Pathol* **139**: 17–23

Shiozaki H, Iihara K, Oka H, Kadowaki T, Matsui S, Gofuku J, Inoue M, Nagafuchi A, Tsukita S and Mori T (1994) Immunohistochemical detection of α-catenin expression in human cancers. *Am J Pathol* **144**: 667–674

Shiozaki H, Kadowaki T, Doki Y, Inoue M, Tamura S, Oka H, Iwazawa T, Matsui S, Shimoyama K, Takeichi M and Mori T (1995) Effect of epidermal growth factor on cadherin-mediated adhesion in a human oesophageal cancer cell line. *Br J Cancer* **71**: 250–258

Su LK, Vogelstein B and Kinzler KW (1993) Association of the APC tumor suppressor protein with catenins. *Science* **262**: 1734–1737

Takayama T, Shiozaki H, Inoue M, Tamura S, Oka H, Kadowaki T, Takatsuka Y, Nagafuchi A, Tsukita S and Mori T (1994) Expression of E-cadherin and α-catenin molecules in human breast cancer tissues and association with clinicopathological features. *Int J Oncol* **5**: 775–780

Takayama T, Shiozaki H, Shibamoto S, Oka H, Kimura Y, Tamura S, Inoue M, Monden T, Ito F and Monden M (1996) β-Catenin expression in human cancers. *Am J Pathol* **148**: 39–46

Takeda H, Nagafuchi A, Yonemura S, Tsukita SA, Behrens J, Birchmeier W and Tsukita SH (1995) V-src kinase shifts the cadherin-based cell adhesion from the strong to the weak state and b-catenin is not required for the shift. *J Cell Biol* **131**: 1839–1847

Takeichi M (1991) Cadherin cell adhesion receptors as a morphogenetic regulator. *Science* **251**: 1451–1455

Takeichi M (1997) Functional correlation between cell adhesion properties and some cell surface proteins. *J Cell Biol* **75**: 464–474

Tsukita SH, Tsukita SA, Nagafuchi A and Yonemura S (1992) Molecular linkage between cadherins and actin filaments in cell–cell adherens junctions. *Curr Opin Cell Biol* **4**: 834–839

© Cancer Research Campaign 1998